# Book Reviews

**Published:** 1831-05

**Authors:** 


### CRITICAL ANALYSES.

Quæ laudanda forent, et quæ culpanda, vicissim

Illa, prius, cretâ; mox hæc, carbone, *notamus*.—PERSIUS.

*The Physiology of the Fœtus, Liver, and Spleen.* By GEORGE CALVERT HOLLAND, M.D., Bachelor of Letters of the University of Paris, &c.—8vo. pp. 229. London: Longman and Co. 1831.

THE physiology of the Fœtus, though first named in the title, concludes Dr. HOLLAND's volume; and the physiology of the Spleen, though at first it stands last, becomes the prior object of discussion: hence it is that with which we shall begin, giving, of course, precedence to some remarks on the preliminary dissertation on the "Causes which have retarded the Attainment of sound Principles in Physiology," which, by the bye, we do not think much to the purpose; for, however well its censures might have suited a former age, certain it is that the present can scarcely, with truth, be accused of preserving "that undue veneration for antiquity, and that servile reverence for great names, which have so frequently prevented the discovery of truth." Of this vice, the existing generation of philo- and pseudo-sophs may surely be acquitted; though to the accusation of its converse, we fear that few could justly plead not guilty. Indeed, we have long viewed with disgust that undue disrespect for any opinion that is ancient; that flippant irreverence for great names which characterizes, in so great a degree, the tyro of our day, when every inceptor-candidate for medical renown becomes a self-created judge of his master's qualifications, and acts as if he thought that an old theory, like an old coat, although it once was good, has been worn out by time, and is worthless, merely because it is old.

Neither can we agree with the essayist, that "the circulation of the blood, the connexion between the powers of the mind and the development of the brain, the exact states of the body which promote or retard the generation of animal heat, and the true mode in which the fœtus is nourished, are secrets which might have been made known, long before the period of their actual discovery, by any man of ordinary capacity: if, instead of being misled by prevailing opinions, he had carefully examined the phenomena which nature presented to his view:" for this mode of reasoning is, as it were, a begging of the question; it is on a par with the arguments of those who, wishing to detract from the merits of Columbus, observed, that, as to the discovery of America, it was nothing to be wondered at; the only wonder was, that it had not been discovered centuries before; for a man had only to point his vessel in a *certain* direction, and sail in an undeviating course for a *certain* time, and then, in spite of himself, he would run his nose against the American continent. The practical answer of Columbus is familiar to every one; and, as he desired his objectors to make an egg stand, so do we desire Dr. Holland to explain the problem of "generation," the physiology of which, we doubt not, will seem quite as simple, and as easy of detection, *when* found by some adventurous navigator in the unknown seas of science, as (he conceives) the circulation of the blood or the nourishment of the fœtus; although we must be allowed to doubt whether a want of respect for antiquity, and any undue inattention to great names, will be the compass that can direct the voyager in safety. For our part, we know nothing more *uncertain* than the so-called *certain* things.

Again, we must enter our protest against the doctrine that "the ancients, though possessing much less general knowledge than ourselves, and not having nearly so accurate an acquaintance with the human frame, were *almost as successful in the treatment of most diseases;*" for a very superficial acquaintance with the medical records of antiquity would suffice to disprove such a damnatory assertion: nay, has not ancient medicine, on the contrary, long become a by-word for credulity? Nor could it be otherwise when, as medical literature informs us, it was found that an incantation, or a votive tablet, were often *as effectual* in the cure of disease as any of the extraordinary means which the then state of physic sanctioned, and which physicians and surgeons generally prescribed.

Neither can we agree that it has been justly asked (although the inquiry was proposed by DUGALD STEWART,) with regard to the present backward state of medical science,

"'What has medicine yet effected in increasing the bodily powers of man, in remedying his diseases, or in lengthening life, which can bear a moment's comparison with the prodigies effected by education, in invigorating his intellectual capacities; in forming his moral habits; in developing his sensitive principles; and in unlocking all the hidden sources of internal enjoyment?'

"Education has certainly done much more to strengthen and improve the mind, than medicine to invigorate and relieve the body." (*Introd.* p. xiii.)

With all due deference to such great authority, we would venture to suggest that the subjects here compared cannot, in the terms made use of, fairly admit the parallel: for the body, when sown, grows up, and arrives at its perfection, in almost every instance, when guided by the unfettered hand of nature. Here there is little or nothing for medicine to do: maternal care is the early education of the body, exercise and food the volumes it runs through and digests; and it would be hard to blame medicine for doing nothing, where nothing is required to be done; its non-interference should rather merit praise. Medicine (using the term in its ordinary acceptation, and as it seems limited in the query,) is only wanted to guard the body against the access of disease, or to restore the corporeal frame to its normal state, whenever any aberrations from that state occur: whatever art does more, it does amiss; as, for example, take the cases of Carib heads, English waists, and Chinese feet.

As well might it be urged, that the artisan has done little towards the improvement of a machine constructed by some eminent engineer, and which has come to him perfect from a master hand, to what he has done by the right application of its powers in the improvement of the fabrics it was designed to make, as that medicine has effected little in increasing the bodily powers of man, lengthening his existence, &c., to what education has done in invigorating his intellectual capacities, forming his moral habits, &c.; which mental acquirements are, in truth, but parallel to the dexterity and agility which the labourer acquires by the constant use of his corporeal members in works of art.

Art is the further education of the body, as science is the education of the mind, and much has been done by both, in the development and proper application of their respective powers: the exercise of walking will strengthen the legs, that of rowing, the arms; the senses of hearing and seeing may be rendered more acute by the exercise of their respective organs, just as the memory may be strengthened, and the reasoning powers rendered more subtle and acute, by appropriate exercise; and, if few labourers could solve a problem, few philosophers could make a mousetrap.

Hence let it be remarked, that education should be esteemed the food and exercise, rather than the medicine, of the mind; and, furthermore, let it be observed, that the schoolmaster, with his lessons and his rod, does no more for the one than the nurse, with her diet and her pastimes, does, less ostentatiously, but not less effectually, for the other.

But, perhaps, it may be argued, that, if education be withheld, the mind will remain puny, weak, and feeble; the mental powers be blighted; and those higher developments, which exalt and characterize the human race, become degenerate and abortive: yet this is nothing more than saying, that the mind may become diseased from the use of improper, or starved by the total want of food, just as the body, under equivalent circumstances, may be weakened, diseased, or starved: nor do we think that certain trades and occupations, deprivation of wholesome food, the use of ardent spirits, &c. more surely affect the corporeal health and strength, deform the limbs, and shorten life, than that certain modes of education, the prosecution of certain studies, the devotion to superstitious systems, the indulgence in the perusal of romantic legends, &c. will weaken, cramp, disease, and oftentimes destroy the mind.

Thus, had the parallel been fairly drawn, i. e. had the question been asked, which has been most successful, the culture of the body or the culture of the mind? which has done most to relieve and remove the infirmities and disorders incident to each, the physician of the body or the physician of the mind? then it would not have been said, that medicine has made but little progress in that which is its peculiar and appropriate province: it has already done much, and doubtless it will do much more, "to invigorate and relieve the body, to remedy its diseases, and to lengthen human life." Have the preservative influences of inoculation and vaccination been forgotten? Has the absence of plague, of dysentery, and the sweating sickness, from this country, not proved beneficial to health and life? Are operations for hernia, and aneurism, and schirrus, of no avail? Ask the late sufferer with stone in the bladder, whether even lithotomy was not a blessing? and ask the present, whether the modern improvements in lithotrity have not disarmed the disease of half its terrors? And hence, to quote our author's words, we do not think that "education has certainly done much more to strengthen and improve the mind, than medicine (taken in as equally confined or extended a sense, has done) to invigorate and relieve the body."

But, enough: it would be tedious even to hint at the improvements in modern medicine, and to enumerate the diseases now subject to our control which were formerly indomitable; let the life-assurance offices be our witnesses that disease is less common, and less fatal, now than formerly: their rapidly decreasing ratios of premium attest, in the intelligible language of £, *s.*, and *d.*, that medicine has affected something towards increasing the bodily powers of man, towards remedying his diseases, and towards lengthening human life, which the sick at least will think can bear more than "a moment's comparison with the prodigies effected by education."

But to proceed. We perfectly admit, indeed we think few will venture to deny, the force of the following truism, asserted with reference to Magendie, viz. that, "had all the writers on physiology and medicine possessed the talents of this distinguished author, and industriously employed them in the study of the same sciences, they would have been more clearly understood and more fully developed;" for this is but tantamount to the assertion, that if there were more labourers who did more work, more work would be done by a greater number of industrious workmen than by a smaller number of lazy ones. Still we think things are better as they are, than, if all physiologists were like Magendie, great as undoubtedly he is; for, much as we admire the talents and respect the industry of Magendie, we should regret to have all our medical philosophers struck from the same die, cast in the same mould; and we think, with our author, that, "if there be one fault (and it is a fault) in this eminent physiologist, it consists in his too confined and exclusive attention to experiments, which sometimes prevents him from taking a sufficiently enlarged view of the animal economy, and from classifying the various facts with which he becomes acquainted, so as to render them practically useful." Phenomena are so often misunderstood, or misperceived, by those who make experiments and observations, and recorded data are so often misconstrued, and misapplied, by those who have seen not, but only have read or heard thereof, that we give in our hearty adhesion to the following sentiments: that,

"However valuable the important discoveries which have been made by a recurrence to experiments, much error has risen from the false inferences that have been frequently drawn from them, inferences which, though really the mistakes of erroneous observation, have been regarded as incontrovertibly correct.

"It is very allowable to doubt the truth of propositions supported by argument alone, but it seems an act of bold temerity to call in question facts, or obvious deductions from them said to be proved by direct experiment; and hence, whilst the former are fearlessly controverted, the latter are generally received with too easy an assent." (*Introd.* xx.)

Let this be constantly borne in mind: it was by no mean authority declared, "that there are more false facts than false theories in medicine."

But a truce to these introductory observations; we must hasten to the physiological essays, which contain matter much more to our liking: indeed, the introduction has so little to do with the matter it introduces, that we cannot conceive why it was printed, further than because that, in some idle hour, it was written.

*"The Physiology of the Liver and Spleen."*

Alas, poor spleen! who has not read its infinite physiologies, and their most excellent ambiguity? What absurdity has it not borne on its back a thousand times? How much paper has it caused to be spoiled; how much time to be wasted; how much ingenuity to be frittered away? Well has the saying here been verified, that, if a man have a hundred sheep, and lose one, he will leave the ninety and nine in the wilderness, to seek after that which has strayed. And what has been the result of all these reiterated attempts to pen that stray sheep, the spleen, within the legitimate fold of physiology? We know not that the digestive or purveying theories are a whit more true than the carrier-like speculation, that the spleen was stuffed into the left hypochondrium in order to make the packing sure; or than that which conceives it created merely to puzzle physiologists; or than that more ancient doctrine, which declared it ill-humour's elaboratory and especial seat: indeed, in support of this latter hypothesis, we confess that an essay on the Functions of the Spleen not unfrequently ruffles our critical equanimity, and hence, for such a temper, the synonyme splenetic would not come much amiss.

The physiology here presented is, however, the most rational and satisfactory that we have met; and our chief objection, which, however, does not apply to the theory so much as to the theorist, is that the idea now propounded as original, has been, in its most important features, familiar to us for some time past. We well recollect the late professor of anatomy and surgery mentioning, in his lectures on Comparative Anatomy, delivered in the theatre of the Royal College of Surgeons in London, that Dr. Hodgkin, of the Borough, had suggested that the probable use of the spleen was as a *diverticulum* for the blood, when, during any sudden excitement of the mind, or undue exertion of the body, it was sent in too full and rapid a current from the heart; and thus to ward off those dangers that might occur from sudden congestion taking place in any organ of vital importance: this to us then original hypothesis was somewhat dwelt on by the lecturer, and it was specifically stated that this theory considered the spleen as an organ of safety, a *diverticulum,* which would receive a large quantity of blood on an emergency, and return it at leisure to the system, when the circulation became more tranquil. And such is, in fact, the pith of Dr. Holland's physiology; only here the original idea has been amplified, and the liver considered as a second *diverticulum,* and subsequently the thymus and thyroid glands, with the renal capsules and placenta, are declared to be further *diverticula* likewise.

But as our author makes out, on the whole, a good case, we will let him state the theme of his discussion in his own words.

"The present subject is one which has always been considered difficult, and the researches of the most distinguished anatomists and physiologists have not hitherto removed the mystery in which it is enveloped. Most of the opinions advanced by the ancients are hypothetical, and those which have been brought forward in modern times, aided by a more intimate knowledge of the animal economy, are either of this character, or too contracted, in theory or experiments, to be regarded as complete and satisfactory.

"I shall not trouble the reader with an enumeration of the different opinions which have been advanced concerning the office of the spleen; for, as I do not propose to attempt a refutation of them individually, it is not necessary to state them. Two of the best books upon the subject, with which I am acquainted, are Dr. Stukely's, published in 1723, and one by Moreschi, Professor of Comparative Anatomy in Pavia, in 1803. The doctrine inculcated in both is, that the spleen is an organ appropriated to the digestive function, supplying blood according to its demands. When the capacity of the stomach is enlarged by the food which is received, it is supposed to press upon the body of the spleen, so as to diminish the accumulation of blood in the splenic artery, and thereby to augment the action of those vessels which are distributed upon the stomach. This view, with slight and immaterial modifications, is the one proposed by both authors. The necessity of a vigorous circulation during the process of digestion can scarcely be called in question. This state of the sanguiferous system is to be regarded as indispensable, and the views which support it are much more consistent than the ideas at present entertained with respect to the efficiency of the nervous system." (P. 1.)

"The authors first referred to having endeavoured to shew that the spleen performs this vital office to the stomach, find a difficulty in explaining how the stomach acts when deprived of this auxiliary; in fine, the difficulty appears so great, that Moreschi gives little credence to the possibility of such an occurrence in the human frame, and observes that this taking place in the inferior animals cannot be considered as any objection to the action of the spleen in the human subject.

'If authorities were required to prove, that the human spleen has sometimes been excised, it would be easy to bring forward instances. Several cases are on record, where part of the organ was excised, and the remainder was returned without injury to the individual; and it is mentioned by authors of credit, that accidents have occasionally happened in which the spleen has protruded and has been removed with safety, the vessels having been previously secured by ligatures. It has also been stated that, in one or two remarkable cases, no spleen whatever was found; and, in many persons, it has been observed so diminutive, as to preclude the idea of its exercising that function with which physiologists had endowed it." (P. 4.)

The subsidiary digestive, the most plausible of the older hypotheses, being thus summarily disposed of, though comparative anatomy would have afforded other assistant refutations likewise, the spleen being in some animals so situated that the distended stomach could not compress it, the author, after a slight allusion to the experiments of Sir E. HOME, and those of MALPIGHI, ASSOLLANT, and others, which shew that the spleen may be extirpated, (Assollant performed the operation on forty-six animals, without impairing their powers, either of digestion, absorption, *circulation*, respiration, voice, secretion, nutrition, locomotion, sensibility, reproduction, &c.) proceeds to examine the hypothesis that the spleen assists the liver in the formation of bile. This, however, is negatived; and the doctrine that even the liver secretes bile from venous blood, is condemned as erroneous likewise.

"The physiology of the liver is almost as little understood as that of the spleen. Much has been written on the function which it is supposed to perform, viz. the secretion of bile; but it is still undecided whether this be effected by means of venous or arterial blood. If by the latter, for what end is so much venous blood directed to the liver? and, if by the former, the difficulty still remains in accounting for the great quantity of oxygenated fluid which is transmitted to it.

"In whatever manner the subject is viewed, according to the established notions of the day, the physiology of the liver is involved in obscurity. Bichat, after enumerating the various uses of the liver, observes, "De toutes ces considérations et de beaucoup d'autres que je pourrais ajouter, on peut conclure, je crois, que le rôle inconnu que le foie joue dans l'économie animale, outre la sécrétion bilieuse, est des plus importans. L'étude de ce rôle est un des points les plus dignes de fixer l'attention des physiologistes." (P. 10.)

How any difficulty can arise, as here stated, with regard to "accounting for the great quantity of oxygenated fluid" transmitted to the liver, we are at a loss to conjecture: it does not seem to us that the hepatic arteries are of a greater capacity than the nourishment of so large an organ might be reasonably supposed to require: indeed, we find, on reference to his Physiology, Mr. MAYO states that the hepatic arteries "are *small* in proportion to the magnitude of the viscus."

When our author considers the question of the secretion of bile from arterial blood, (which we do not mean to deny,) as settled in the affirmative by the case recorded in the Philosophical Transactions, by Mr. Abernethy, where the vena porta terminated in the cava inferior, without its contents having previously circulated through the liver, and the illustration derived from comparative anatomy, in which the liver of the Mollusca is supplied with blood from the aorta, he should likewise have noticed that, in the lusus, the "hepatic artery" is described as "*being larger than common;*" and also the counter observations of Simon, who found, in his experiments on pigeons, "that, when the hepatic *artery* is tied, the secretion of bile continues; but that, if the veins of the porta and the hepatic canals be tied, no trace of bile is subsequently found in the liver." It should likewise be recollected, that "the capillary branches of the hepatic arteries and the vena portæ communicate very freely, so that a fine injection passes very readily from the one system into the other; so that, as Mr. Mayo concludes his chapter on this subject, "we are, perhaps, bound to attribute the secretion of bile, in human beings, both to the artery and the vein."

But our author is rather "disposed to infer that all secretions are to be ascribed to the qualities of arterial blood, and consequently that bile is derived from this fluid; though he does not imagine that the liver performs no other function than the secretion of bile."

"From what has been previously explained, it is evident that the hepatic artery is regarded as the source of bile: and believing the production of this to be only one function of the liver, it is my intention to account for the great quantity of venous blood transmitted to it and the spleen. Both organs are well adapted to receive a great share of sanguineous fluid, whether we consider the texture as composed of blood-vessels or of cells.

"The function of the spleen, as well as that of the liver, independently of the secretion of bile, is considered a diverticulum of the system. If the veins which form the vena porta had passed directly to the vena cava inferior, a thousand accidents, arising either from mental or corporeal disturbance, would have continually placed the life of an individual in imminent danger. Every passion, whether of an exciting or depressing character, and every general and local disease, if severe, whether chronic or acute, would have been liable to have deranged the lungs and heart. I have already endeavoured to shew, that passions, of a depressing nature, bring the blood in greater quantity than usual from the inferior and superior extremities, and also from the surface of the body, to the chest; and I have also stated, that the abdominal viscera participate in this engorgement. Since the body is continually liable to such changes, baneful effects would follow, unless nature had provided organs, whose situation, function, and organization, enable them to diminish the burden imposed upon those, whose constant and almost regular action is indispensable for the maintenance of life. This object is beautifully answered by the liver and spleen. The organs within the chest must be regarded as possessing vital functions. If the lungs were surcharged with blood, or in a condition approaching to it, the properties and distribution of this fluid would be immediately disordered, and with this primary derangement every part of the body would quickly sympathize.

"The liver and the spleen, from being placed close to the thorax, are calculated to relieve the congested lungs and heart, or rather to protect them from sudden and violent commotions; and are also favorably situated to protect, in the same manner, the stomach, whose action is scarcely less vital." (P. 18.)

On these paragraphs our observations must be short: they contain a summary of the entire essay; but it does seem to us an error to state, that *a great quantity of venous blood is transmitted to the spleen:* indeed, were it so, half the beauty of the hypothesis, and more than half the efficiency of these diverticula, would be annihilated; for it must be confessed that the chief injury to vital organs will be expected to arise from inordinate action of the heart, and, therefore, that, to the arterial system a diverticulum would be, if not more so, at least as necessary as to the venous. This, in truth, it seems to find in the spleen, which regulates the supply of arterial blood to the other abdominal viscera, and lessens the effects of the heart's sudden and inordinate action;* as the liver, by being a reservoir at the other extremity of the sanguineous system, will, in like manner, moderate the current, and regulate the supply of venous blood to the heart and lungs.

"The spleen is, by some, supposed to be formed of vessels; by others, to be chiefly composed of cells; but, whichever opinion we adopt, it is manifest, that its structure is particularly well formed to allow of considerable congestion or enlargement. In those instances, in which the circulation is very much disordered, and in which we have determination of blood to the internal organs, the liver and the spleen are occasionally so much augmented in size, that they cannot be supposed to contain less than many pounds of blood. If these organs had been absent, or incapable, from structure or situation, of receiving an extra proportion, this quantity would have been added to that which is already retarding the action of more vital organs, the consequence of which would be the total cessation of life. We may complain of the enlargement or disorganization of the liver and spleen, but we must remember that, although we occasionally suffer from the diseased conditions of these organs, we

* May not the pain in the left side, which occurs in running, be attributed to over or sudden distention of the spleen?

are preserved from many acute affections, which would seldom allow man to arrive at maturity, unless the constitution were endowed with instruments fitted to bear oppression. If we consider the temperaments most subject to derangements of these parts, the diseases which affect very much the internal viscera, and the influence of external agents upon the body, we shall probably observe more clearly the consistency of the view proposed to explain the functions of these organs.

"Of all the temperaments, the melancholic is by far the most frequently characterized by disease or augmentation of the liver and spleen. Indeed, so common is it to find these conjoined, that it has been imagined that there exists a necessary connexion between them.

"The greater part of the ancients supposed, that these organs were the seats or causes of this temperament, and although it is impossible to grant that any viscus or viscera of the abdomen can fashion the peculiar constitution of the mind, still the universal language of mankind proves, that these organs were generally large or diseased in individuals of this temperament. We very rarely find persons so constituted take constant exercise; their habits, for the most part, are sedentary, and instead of enjoying the gaiety and hilarity of convivial parties, they generally prefer solitude, or are occupied in brooding over real or imaginary evils. In the chapter on the Physiology of the Passions, already alluded to, the manner in which the body suffers from a disorder in the powers of the mind is fully discussed; and as the feelings of melancholy are considered to operate in the same way as those which were referred to the division of mental sedatives, there is little further to add on the present occasion.

"A life of inactivity, or one abounding in disagreeable sensations, tends to determine the blood internally, and those organs which are best adapted to bear this determination, or state of congestion, will suffer to the greatest extent. The liver and spleen being formed in every respect to receive the principal share, they will necessarily exhibit symptoms of derangement or disease, as if they were the only disordered organs; but in this state of the system we also remark, very frequently, if not constantly, acute headach, palpitation of the heart, sometimes cough and difficulty of breathing, or aberration of the mental faculties: and unless the two abdominal viscera had been so constituted and placed, the whole train of the latter effects would have become too prominent for the existence of the animal economy. If these principles are allowed to be correct, the treatment of nervous diseases must be considerably modified.

"From the variety and importance of the abdominal organs, diseases are frequently occurring in them. Congestions, or inflammations, either remaining local, or exciting general fever, or producing their ordinary consequences, such as adhesions, disorganizations, dropsies and tumors, disturb the regular course of the circulation, and very much disorder the functions of the heart and lungs. But how much more would these organs be oppressed, if the liver and spleen had been incapacitated, either from structure or functions, for performing the office for which nature is supposed to have intended them! If each had any other exclusive part assigned it, to secrete, for example, to assimilate, to propel, or to endue with sensibility, or motion, it would be impossible to conceive, that any one of these functions could remain amidst such constant disorder, or disease: and if this argument be allowed, it is clear that the more vital or necessary organs of the system could not withstand the oppression arising from the determination or congestion of blood.

"Intermittent fevers, and particularly those of the quartan type, are, of all agents, the most powerful in disturbing the abdominal organs. In these the liver and spleen are occasionally observed of immense size, occupying the two hypochondriac regions, and extending over a considerable portion of the abdomen towards the hypogastrium. It would seem that these large organs were the seats of the disease, but as they have little influence in causing or exciting a predisposition to it, we must look to other sources for its origin; and, if we reflect for a moment on the quantity of blood accumulated in these two viscera, we shall be almost under the necessity of acknowledging that, if this had been distributed equally among the heart, the lungs, the brain, and the abdomen, the powers of life would have been destroyed.

"The various organs enumerated are not deprived of their usual share of the sanguineous fluid, during the congested condition of the abdominal organs, but actually possess more, and therefore a farther increase would be fatal to their action." (P. 20.)

"In speaking of the influence of passion, I alluded to several cases of jaundice, mentioned by Bichat and Georget, as the consequence of depressing feelings of the mind; and such a phenomenon, according to the explanation given, shews the origin of the symptom, and the liability of the liver to congestion. This is an occurrence by no means rare; but the frequency and severity of this condition, arising from mental emotions, demonstrate the necessity of such an organ. If it were absent under such circumstances, the lungs would immediately be surcharged with blood; if they were able, for a short period, to continue their operations, these would be so feebly and imperfectly performed, that death would speedily take place, either in the heart or the brain. Is it possible, then, to suppose that the liver and spleen have no functions, when they conjointly contribute to preserve the constitution from almost instantaneous destruction, or acute diseases, whenever the equilibrium of the circulation is deranged." (P. 27.)

"Having now briefly considered the importance of the liver and spleen, as diverticula to preserve from disease, or to alleviate its severity, it is scarcely necessary to observe, that it is easy to explain the possibility of the spleen being excised in man, and in the inferior animals, without detriment to either, except from inflammation almost inseparable from the operation. If it be removed, and the individual recovers his wonted energies, these may exist for a series of years; because their exercise does not depend upon the spleen, but upon the proper action of organic laws, which are equally independent of the same organ in the undisturbed state of the system. Its office is not to contribute, every moment, to the maintenance of life; but only, on trying occasions, to develope the full powers of its functions, and, in conjunction with those of the liver, to protect the vital principle from destruction." (P. 29.)

We must now conclude our review of this, the first Essay in Dr. Holland's volume: it has run to a greater length than we proposed, but it contains some curious and interesting matter. He shall sum up the argument in his own words:

"Wherever a sanguiferous system exists, and is liable to be affected by mental or physical causes, or by both, there is evidently an absolute necessity for organs capable of receiving the greater portion of the blood, which is occasionally determined internally in an undue proportion. It therefore seems consistent with analogy and facts, to regard the liver and the spleen as diverticula, though in ascribing this office to the former, I by no means intend to question its importance as a secretory organ." (P. 37.)

Since writing the above, we have referred to our own memoranda, and have likewise been favored by a friend, who took shorthand notes, with a reference to his, and we find that the lecture to which we have alluded was delivered by Mr. Green, 12th April, 1828, in which, along with a summary detail of the principal ancient hypotheses, and the more modern opinions of Haller, Haighton, Cline, Tiedeman, Leary, Lascelles, Cooper, himself, and others, he introduced Dr. Hodgkin's theory in nearly the following words: "One of the most ingenious uses, as a diverticulum, was promulgated by my friend Dr. Hodgkin; and, if I understand him rightly, he conceives that the spleen acts as a diverticulum to the blood, under any disturbance of the vascular system; that it is, as it were, an organ destined to receive a congestion of blood; that, under many circumstances, there would be an undue disturbance of the vascular system, and hence an irregular distribution, perhaps fatal congestion, of blood, were not the spleen to receive the overflow, and prevent the occurrence of any ill effects; or, to use the words of the Doctor, 'it tends to obviate any sudden disturbance that might arise from the temporary disproportion of the capacity of the vascular system, or any part of it, to the fluids which circulate therein."

From this it will be seen that Dr. Holland has been anticipated in the grand feature of this physiology, viz. the doctrine that the spleen acts as a diverticulum to relieve the circulation, whenever unduly excited or increased; and, although an important addition, still the considering the liver as a second diverticulum is but an amplification of the original hypothesis, just as the following physiological inquiry into the Uses of the Spleen, by Mr. JOHN TUSON, published in a late number (171) of the Medical Gazette, is but an illustration of its use in one case out of many in which the circulation is affected, although neither of the Drs. H. are in it referred to: indeed, it is a combination of their theory with those of HAIGHTON, LEARY, LASCELLES, and others.

It appears that this viscus (the spleen) is capable of containing a very considerable quantity of blood, and it incontrovertibly appears, likewise, that the sanguiferous system is only capable of containing a certain proportion; so that it follows that a considerable increase of its volume must necessarily be the result of the chyle being conveyed into the blood-vessels.

It is perfectly unnecessary to the philosophic inquirer to point out the serious ills which would ensue, were not some precautions taken to prevent the over-distention of the sanguiferous system. He must be well aware how carefully nature, in the formation of every part of the human frame, has provided and guarded against any evil that could by chance assail it. Taking a comprehensive view, marking the dependence and accordance of things, we shall find nature has not been less attentive in this respect; and we shall see that our all-wise and ever-provident Protector, who has so harmoniously constructed every part of the human frame, has appropriated this viscus as a reservoir for the reception of this superfluous blood; thus obviating these difficulties. Contemplating this viscus, viewing its structure, and observing how admirably well fitted it is for this important purpose, it is impossible to suspend our assent to the doctrine above stated. Here we have one of the grand purposes to which this important viscus is adapted; for the discovery of which the public are indebted to the persevering industry of Dr. DOBSON.* His experiments incontrovertibly prove that the spleen is replete with blood when the chyle is conveyed into the system; and, on the contrary, that it is empty when the office of digestion is fully completed. To this work we must refer the reader: it would be injustice in us to recite it. His experiments and observations are ingenious, and highly inte-

* See a Review of Dr. DOBSON's work: London Med. and Phys. Journal, October 1830, p. 356.

resting; so much so, that they have induced us to make the foregoing observations illustrating this theory. Let it not be imagined, however, that we consider this the only important purpose to which this viscus is assigned: there are other purposes, equally essential to the welfare and well-being of the animal economy, which the spleen is destined to contribute to, one of which is the formation of the bile. The pathological derangement of the spleen fully elucidates this doctrine; and what still further confirms this opinion is, that, whenever the spleen is removed from the body by dissection, the liver becomes swelled and disordered, makes a less quantity of bile, and of a dark brown colour, while the animal is perpetually troubled with flatulence and indigestion."

(To be concluded in our next.) p. 543.

---

*Cases of Lithotrity, or Examples of the Stone cured without Incision; followed by a Description of the first Symptoms of the Disease.* By Baron HEURTELOUP, Doctor of the Faculty of Medicine in Paris.—8vo. pp. 54. London: T. and G. Underwood, 1831.

LITHOTRITY, one of the most important improvements in modern surgery, has already occupied many of our pages, and its merits been discussed in several numbers; for we have considered it our duty to recur to this subject as frequently as any notable progress has been made in perfecting the apparatus, or in the application of the various instruments to the destruction of calculi in the human bladder; and this little pamphlet, the harbinger of a larger work, will prove that we have not been too sanguine in our anticipations of the benefits which may from this operation be derived. Fourteen successful cases are here recorded by Baron HEURTELOUP, and one of these patients twice relieved: now, fourteen cases of stone in the human bladder, cured in little more than eighteen months, by one individual, in this country, not computing those which have been successfully treated here by Mr. COSTELLO and other surgeons, or those more numerous ones which, during the same term, have been relieved in a similar manner by continental surgeons, must form a subject of no slight philanthropic congratulation.

We would fain transcribe the whole of these fourteen cases, but as several are, in most of the essential points, similar to each other, we shall content ourselves with selecting from them the most interesting and important features.

"It is," indeed, as the author, in his Introduction says, "Important that this system of operating, so beneficial to mankind, should not only be known to exist, but also that it has already been put into practice with success in England, for the cures effected in a country which is to derive benefit from this novel and effectual means, afford a more striking conviction than those obtained in a distant country; it is with this intention solely that I limit myself to the publication of the first cases which I have obtained in England, exclusive of those much more numerous instances which I have happily treated in France." (P. 1.)

The first case recorded is that of Mr. Wattie.

"Mr. Wattie, formerly a seaman, sixty-four years of age, residing at No. 78, Upper Ebury street, Pimlico, was presented to me by Mr. White. In three applications of the 'perce-pierre,' which took place on the 24th and 30th July, and on the 20th August, 1829, two calculi were destroyed, which might have been from six to eight lines in diameter.

"There was nothing very remarkable in this case, except a violent contraction of the bladder, which Mr. White and myself proved to be, as nearly as possible, equal to that which would result from a pressure of thirty-seven pounds on an animal's bladder distended with water. Mr. Wattie's case is, however, to myself rendered interesting, from his having been the first patient treated by lithotrity in England; and I feel much gratified in having this opportunity of expressing my obligation to Mr. White, for his liberality in confiding to my care the first patient operated on in this country, with the instruments used in lithotrity, and for thus laying the foundation of that success which has accompanied my subsequent progress." (P. 6.)

In the case of Major Moore, of Dublin, who, in two operations, had a stone evacuated of about ten lines in diameter, which had previously been attempted to be extracted by the curved forceps, but without success.

"The only circumstance worthy of remark is that the stone, when once forced into the cervix of the bladder by the urine, was retained there for a considerable length of time; this threw the patient into a continual state of spasm, and produced a catarrh of the bladder, which ceased entirely when the stone was extracted." (P. 8.)

Case III. "Admiral ***, of Exeter, sixty-four years of age, was placed under my care by Mr. White. He had, for several years, been afflicted with a stone, which was about fifteen lines in diameter, when I sounded him; it was spherical, composed of uric acid, and very hard. The prostate gland was enlarged to such a degree, that it obstructed the free passage of the urine, which was thick, and deposited a considerable quantity of muco-purulent catarrh, and which the patient could not eject in a full stream; he felt a desire to make water nearly every half hour, and the expulsion of the urine was attended with acute pain; the bladder was very uneven, and intersected with septa; beneath the enlarged prostate there existed a considerable pouch which lodged the stone. Notwithstanding these disadvantages, I undertook to destroy the calculus. It was first broken down with the 'évideur à virgule,' and afterwards comminuted with a common three-branched instrument, with only one hooked extremity, on account of the irregularity of the bladder. As the cavity, situated under the cervix of the bladder, hindered the stone or fragments from being either felt or seized, while the patient was placed horizontally, it was frequently found necessary to operate on him in an inclined position, which was easily obtained by means of a rectangular bed. The case before us proves the importance of this position. The admiral being unable to make water in a stream, on account of the enlarged prostate, it followed that he could only pass the powder, and no fragments; this circumstance induced me to construct an evacuating sound with which the patient was enabled to void large pieces of calculi.

"The admiral left London in perfect health in February, but wrote me a letter about five months after his arrival at Exeter, stating that his former sufferings had returned. I desired him to come up to London immediately, sounded him, and discovered a calculus, nearly as large as the one of which he had just been relieved. The stone was comminuted, and the fragments brought out in the same manner as before. The admiral very naturally thought that this second calculus must have had for nucleus a fragment of the former one, left in the bladder. I was of the same opinion, although Mr. White and myself had sounded the patient three or four times after the last operation, without finding the smallest remnant of calculus; but a careful examination of the fragments of the second stone, convinced both the patient and myself, that it was quite a new formation, for it was white, and purely composed of triple phosphate, whilst the first one was red, and entirely formed of uric acid: if a fragment of this stone had remained in the bladder, it would immediately have been recognized.

"It is probable that this patient will be again afflicted with the stone, since, in his case, it forms so rapidly. I warned him of it before he left, in order that he might not allow the calculus to attain any magnitude. The admiral can only be completely cured, when, by a process of nature, his constitution changes; for as, from a sound state of health, he became subject to form calculous concretions, so, from a tendency to make stone, he may recover his primitive constitution; he has therefore every reason to hope for a perfect recovery, if he has not already obtained it, and if, as I suppose, the disease has been removed from the prostate and bladder in consequence of the extraction of the stones.

"The admiral's case is, above all others, suitably adapted for incontestably proving the importance of lithotrity. The patient being naturally disposed to form vesical calculi, must repeatedly have undergone the operation of lithotomy, and every time have endangered his life, had not lithotrity come to his assistance, and afforded relief, without exposing him to any danger whatever." (P. 9.)

Case IV. Mr. Duncan, of Worcester, fifty years of age, had a uric-acid calculus, of about an inch in diameter, which was fractured with the "trois branches à virgule," and the fragments comminuted with a "perce-pierre." This patient had an "extreme sensitiveness of the bladder, which was in a great measure subdued by daily introducing bougies for some time before the operation. This case is remarkable for "the immediate cessation of a blennorrhœa, which existed at the same time as, and was, without doubt, the consequence of the stone; for, as soon as it was extracted, the blenorrhœa disappeared."

Case V. " Mr. Stutcliffe, a farmer, residing at Thortle Bowers, Yorkshire, fifty-five years of age, having read an account of Mr. Wattie's case in the medical Journals, came to London, and confided himself to my care. Two oval stones were discovered in this patient's bladder, which were smooth, and disposed in alternate layers of phosphate of lime and uric acid; they were about twenty lines in length, and from ten to twelve in thickness; the bladder was capacious, but very contractile, and the urethra was large. Notwithstanding the size of the calculi, I considered this a suitable case for lithotrity; and the patient was subjected to the action of the 'perce-pierre,' which was employed in order to weaken the stones by a few perforations, so that the 'brise-coque' might afterwards act with greater effect. It is to be regretted, in this case, that there was not at that time in my possession a 'trois branches à virgule' sufficiently large to fill the patient's urethra, for with this instrument one operation would have reduced the stone to a favorable state for the action of the 'brise-coque,' which the 'perce-pierre' required three or four applications to accomplish. In eight 'séances,' however, Mr. Sutcliffe was relieved of the large quantity of stone which his bladder contained.

" This case clearly proves the importance of the 'brise-coque,' for after every application of this instrument, the patient voided a large quantity of detritus.

" The only thing to which I shall direct attention in this case is the size of the calculi, the detritus of which, when collected, weighed an ounce and a half." (P. 13.)

Case VI. " Mr. Rodgers, of Limerick, sixty years of age, was recommended to me by Mr. Green: he had been suffering from the stone for more than a year, or what is more correct, he observed at that time the first symptoms of vesical calculus. By means of the sound a stone was discovered, which might have been from ten to twelve lines in diameter. It was attacked twice with the 'perce-pierre' with little or no effect; on account of its smoothness and flattened shape, it escaped from the grasp of the branches as soon as the perforator was put into action. This circumstance induced me to employ the 'brise-coque,' with which, in one operation, the stone was broken; and in another, the portions of calculi which remained were reduced into powder, and fragments sufficiently small to be carried through the urethra.

"A fact worthy of notice in this case is, that with the 'brise-coque' was accomplished, in two operations, of short duration, what the 'perce-pierre' could not have performed in a far greater number of applications.

"This cure might very probably have been obtained by means of the 'perce-pierre' alone, but only by repeatedly submitting the calculus to the action of the instrument; by dint of chipping the stone, and breaking off small pieces, it might at last, perhaps, have been comminuted, but only after an exceedingly long and painful treatment. In this case, therefore, the 'brise-coque' proved essentially useful. The orifice of this patient's urethra was also very much contracted, I was consequently obliged to make an incision of the little membranous fold which produces this contraction." (P. 15.)

Case VII. "Mr. Goldsmith, solicitor, about thirty years of age, residing at Watford, was presented to me by Mr. Bransby Cooper, who, having sounded the patient and discovered a stone, advised him to have recourse to lithotrity, which mode of treatment Mr. B. Cooper thought advantageous, on account of the age of the patient, his good constitution, and the favorable state of his bladder. The smallness of the stones in Mr. Goldsmith's bladder rendered the application of the 'perce-pierre' advantageous. With this instrument two or three small calculi were immediately seized and destroyed; but several times, one of them, although secured by the branches, eluded the grasp as soon as the drill was put into action. This clearly indicated that the stone was flat, and I resolved to destroy it with the 'brise-coque,' with which the flat calculus was immediately seized and crushed.

"From this time every symptom indicative of stone in the bladder disappeared, and the patient recovered." (P. 17.)

Case VIII. "A Greenwich pensioner, about forty-eight years of age, consulted Mr. Dobson, surgeon to the Hospital, for a blenorrhœa. This gentleman having sounded the patient in order to discover what might be the cause of a discharge, for which no reason could be assigned, found a large stone in the bladder, and did me the honour to send for me, and put the patient under my care.

"The stone being spherical, and very large, it was thought right to commence the operation with the apparatus known by the name of 'évideur à forceps.' With this instrument, assisted by the 'pince-servante,' which then only served as indicator, the calculus was immediately seized, and firmly secured; for about ten minutes the eccentric excavator worked on its interior, but this was not sufficient to reduce it to fragments. The stone being formed of uric acid, and disposed in layers of different density, though they were all exceedingly hard, did not yield so readily to the action of the excavator as those calculi which are less hard, and, above all, of a more regular grain; all the centre was, however, wasted away by the action of the instrument.

"The two following operations were performed with the 'virgule:' with this instrument a large quantity of detritus was obtained, although the stone was only attacked four times.

"Although the patient had voided many fragments, and a great deal of powder, the fourth operation convinced me that there still remained in his bladder a large compact mass, in which there were several openings formed by the 'virgule,' and a central excavation resulting from the action of the 'évideur à forceps.'

"The excavating instrument being of little avail against the excessive hardness of this calculus, I thought that by percussion I should more speedily determine the fracture of this mass, which, from being hollow, was more likely to be broken by these means than by any other. The excavator could not now act, on account of the irregular interior of the stone, and the perforator was useless, from its tendency to fall into the numerous holes which had been previously made, and from thence into the hollow centre, where there was no substance for it to act upon.

"It was consequently thought expedient to endeavour to break this hard concave mass by percussion; at the next operation the shock of a hammer was communicated to the stone by means of a drill, placed between three very strong branches: in a few strokes the stone was reduced to fragments.

"The 'perce-pierre' was employed twice, to reduce the fragments into smaller pieces, which were afterwards speedily comminuted with the 'brise-coque.'

"This case is remarkable in many respects. In the first place, since this patient was operated on publicly, I thought it right to apply, successively, all the instruments of lithotrity which I make use of, in order to shew the action of each one. The 'perce-pierre' was employed to prove that, although a pretty good instrument in certain cases, it had many imperfections, to obviate which I invented the 'brise-coque:' the immense advantage of this instrument, as much with respect to seizing as destroying the fragments, was clearly pointed out. * * *

"This is the first case in which percussion with a hammer was ever employed to obtain the rupture of the shells of calculi, which the excavators could not reduce to fragments.

"The gentlemen who saw this mode of proceeding could not but think it rather extraordinary that a hammer should be made to act in the bladder, but they were also unanimous in their opinion, that in many cases it might be of the utmost importance. From this cure, then, we may date an improvement in lithotrity.

"When this patient applied to Mr. Dobson, it was to consult him for a discharge which had lasted some time; but the pensioner himself had not the slightest suspicion of the existence of a stone in his bladder, having never experienced the pains, nor observed the symptoms, which it generally produces: we may therefore say that, in this case, the calculus was discovered almost by chance. This circumstance is curious, but not so uncommon as is in general imagined, and it deserves attention; for it proves that lithotrity will frequently relieve patients afflicted with large stones, although it has been said that, when this mode is generally known, there will only be small calculi to destroy; but it is evident that they must sometimes become large, since persons can retain them a very long time in their bladder, without being aware of their presence. I have also to add, that this patient frequently indulged in drinking spirituous liquors during his treatment: this leads us to the conclusion, that lithotrity is accompanied with very little danger, since, by its means, a patient was relieved of a large calculus, and restored to health, notwithstanding these deviations from a proper diet." (P. 18.)

CASE IX. is that of a baronet, who, in three applications of the "perce-pierre," had a calculus, ten lines in diameter, reduced to powder and fragments small enough to be voided by the urethra. This patient had violent contraction of the bladder, which occasioned some difficulty; but this was so readily overcome, that, "after every operation, he returned home on foot, in the same manner as he came, and presided at his own table, in the midst of his friends. This circumstance is mentioned, because it is curious that a person, successfully treated for the stone, should have been all the while living with his family, and that they should be ignorant of what was going forward."

CASE X. That of Mr. Castle, surgeon to the county of Clare Infirmary, sixty-two years of age, is interesting from the following circumstance:

"The calculus was first attacked with the 'perce-pierre,' which I thought might be employed with advantage in this case; but the stone being smooth and flatter than it had first appeared to be, escaped from the branches as soon as the drill pressed upon it. In five applications of this instrument the stone was not once perforated, its outside was grazed, and many small pieces were broken off, one of which evidently corresponded to the edge of a flat stone.

"This was sufficient proof that the action of the 'brise-coque' would be necessary; for to destroy a calculus of this shape, the 'perce-pierre' would require a longer and more painful treatment than the patient could bear.

"In three operations, a stone, which had resisted the action of the 'perce-pierre,' was reduced into fragments and powder.

"This case is important, because Mr. Castle, who is a surgeon, confided himself to my care, in order to find relief by my method. Let such a choice then call attention to that system, which, in the present case, has proved so clearly the insufficiency of the 'perce-pierre,' and the importance of the 'brise-coque;' for let it be remarked that this instrument was only employed when we discovered the almost utter impossibility of curing the patient with the 'perce-pierre.'" (P. 26.)

Case XI. Mr. Archer, aged fifty-two, felt inconveniences occasioned by stone in the bladder; but the existence of calculus could not be decidedly ascertained by ordinary sounding, for "the bladder was irregular, covered with cells, and, during its contractions, it was intersected with fleshy columns, between which the stone might conceal itself, and be secure from any contact with the sound:" but, on the introduction of the perce-pierre, with only one hook,

"Notwithstanding these inequalities, a fungous and varicose state of the cervix, and a high degree of contraction, a small round uric-acid stone was seized, and reduced to fragments and powder.

"This case is interesting for various reasons: it proves that a calculus cannot always be detected by means of catheterism, and that an instrument of lithotrity is not only better adapted than a sound to discover a calculus, but that a surgeon may, in some cases, convert a simple examination into an immediate operation.

"Mr. Archer's case is also curious, from his having had a bladder with a varicose neck, which swelled to such a degree after the introduction of the instrument, that it presented an almost insurmountable obstacle to the expulsion of the urine; for four or five days the patient could not pass a drop of urine without the assistance of a catheter, which was introduced daily, and sometimes more frequently, to empty the bladder: it had the two-fold advantage of bringing out the water, and enabling the patient to expel his fragments immediately, and without the least difficulty. In the course of a few days, he was able to make water in a full stream, but for a fortnight he continued to pass a little glairy mucus." (P. 29.)

Case XIII. Captain Armstrong, sixty-four years of age, had two calculi, one of which was oval, and this was broken by the virgule; the other was too flat to be thus comminuted, and "required the action of the 'brise-coque.' With this instrument, in three applications of four minutes each, a calculus, which had been refractory to all the other instruments, was speedily reduced to fragments and powder."

Cases XIII. and XIV. are very interesting: for the detail of them, we must refer to the work. In these instances, the Baron operated before several of our first surgeons.

The concluding remarks are of much practical importance; we therefore give them in the author's words.

"These are the first operations which I have had an opportunity of performing since my arrival in London; they have all been attended with success, notwithstanding all the difficulties I had to surmount in the treatment of these patients, whose cases were all of so different a nature. I found it absolutely necessary to modify and vary the mode of treatment for each one, either in what regards the course to be followed preparatory to the operation; or else in what relates to the circumstances attending it; or, lastly, as concerns the instruments themselves, which ought certainly not to be the same for destroying stones differing in size and shape, and consequently more or less favorably adapted to the power of differently constructed instruments.

" From these operations we may infer many important conclusions for lithotrity; among which we have selected the following:

" Since large and sometimes numerous calculi have been comminuted, and the patient restored to health, it follows: that lithotrity can be applied in cases of large and numerous calculi.

" Since many of the patients in question have recovered, notwithstanding a considerable catarrh, and great sensitiveness of the bladder, it follows, that lithotrity can advantageously be had recourse to in cases of diseased bladder.

" Since many of these patients have been restored to health notwithstanding a large and swollen prostate, we may conclude that a considerable enlargement of this gland is not an absolute hindrance to the operation of lithotrity.

" Since many of the patients were unable to expel their fragments, and we succeeded in bringing them out of the bladder by means of the evacuating sound, it proves that the impossibility of voiding the fragments does not render lithotrity inadmissible.

" Since flat stones have been crushed with facility by the 'brise-coque', we may infer that calculi of this shape are no longer refractory to the action of the instruments of lithotrity.

" Since the facility and rapidity with which a patient is cured, is in direct proportion to the small size of the calculus, it follows that the operation of lithotrity is far more advantageous when performed at the commencement of the disease.

" Since none of the patients confided to my care have had an attack of fever, during their treatment, since the greater part of them were operated on at my house, and since all experienced so little pain as to allow them to pursue their business more or less, we are led to this important conclusion, that the operation of lithotrity is not attended with any danger, when it is performed in proper time, with necessary care, suitable instruments, and sufficient experience." (P. 39.)

The concluding popular digest of the symptoms of stone in the bladder, although well fitted for the purpose for which it is designed, viz. that of leading patients to detect calculi in their earliest stages of formation, must be already familiar to the profession, and hence cannot need recapitulation here.

In reviewing the rapid progress towards perfection which lithotrity has made, it is scarcely possible for any of those philosophic surgeons who have been so meritoriously engaged in its invention and advancement, not to feel very much (perhaps sometimes unduly) elated with the success which has crowned their efforts, and partial to those instruments they have respectively constructed or improved; still a grateful self-complacency, as well as an honourable rivalry, may exist, without any sinister attempts being made to depreciate the achievements of their compeers, or lauding egotistically their own; without continually, and on all occasions, declaring that it was *"I"* who first did this, and it was *"I"* who invented that; for credit is always given in an inverse ratio to its assumption. These hints are thrown out without any particular application: indeed, they equally refer to the disputants on either side of the question, to which there should be, and which, we hope, will soon have but one. Such practices savour not a little of charlatanism, and their ultimate effect will ever be directly opposite to that which they are designed to produce.

---

*An Introduction to Medical Botany: comprehending the Elements and Terminalogy of Botany, the Linnæan, Artificial, and Natural Systems; a comprehensive Arrangement of Medical Plants, and a Table of English and Linnæan Names. Illustrated with coloured Figures. An improved Edition, containing an Arrangement of the Medical Genera, according to Jussieu, and an Analytical Table of the Properties of Medical Plants.* By THOMAS CASTLE, F.L.S., of Queen's College, Oxford; Professor of Materia Medica to the Eclectic Society, &c.—16mo. pp. 194; three Plates. London: E. Cox, 1831.

THIS seems a somewhat grandiloquent title for so small a volume: indeed, it should rather be considered as the table of contents. Of the previous editions of this work we have already made favorable mention; for, notwithstanding its errors both of omission and commission, it is a useful Manual; and to us it is truly pleasing to find that so much information can be conveyed in so few words, and compressed into so small a space. Of course, many subjects are slightly, some, we think, too superficially treated; still it is calculated, in a great measure, to fulfil the purpose for which, we presume, it is designed, viz. not to supersede the necessity of lectures and of large works, but to recall to the mind of the medical student, in a convenient and contracted form, those facts and doctrines with which he is more especially concerned; the principles of which have elsewhere been explained, and the proofs of which have been elsewhere learned.

There are, however, some blemishes (we had almost said, serious faults,) that should not have been suffered to disgrace this "improved edition." Some few of these, out of regard to a really valuable work, we beg to submit (along with various typographical and minor errors passed over in silence,) to the author's attentive examination; and this we consider our more especial duty, as they can be easily corrected, and as our previous commendations may have placed the book in the hands of many students.

Page 17. Anthemis nobilis should not have been given as an example of a *scape,* for its stem bears both leaves and flowers, and is consequently a true *caulis.*

P. 27. The fruit of Punica granatum is not, as here stated, a *pome;* neither does it agree in structure with the author's own definition, of which it is given as an example. The vulgar name *Pome*granate, like that of Juniper *berry,* although it might mislead a tyro, should not have caused a professor to cite them as examples of Pome and Bacca: indeed, not one of the examples here given agrees with the author's definition of a berry, (same page.) So much has been learned since the time of Linnæus, that we cannot but lament that most of the definitions in the "Elements" are founded upon an old and imperfect physiology, which now is all but obsolete; when the modern doctrines might have been expressed in quite as few words, without danger of misleading the student.

In the "second Part," which relates to the Linnæan artificial system, there are some inexcusable inaccuracies: e.g. the eleventh class is said to be one of those, the characters of which "are established on the *number* and *insertion* of the stamens, as being attached to the receptacle or to the calyx, and corolla," (vide p. 49,) "Dodecandria twelve to nineteen (stamens) to the receptacle;" and again (p. 63) this erroneous definition is repeated: "Class XI. Dodecandria, comprehending those plants which produce flowers with from *twelve to nineteen* stamens inserted into the receptacle;" whereas, on the contrary, it is notorious that, in this class, as in the ten preceding ones, the insertion of the stamens is a matter of indifference: some dodecandrious plants, as Bocconia, Macleaya, &c., being hypogynous; others, as Lythrum, Peplis, Sempervivum, &c., being perigynous; and others, again, as Asarum, being epigynous. Indeed, most paradoxically have the illustrations been selected, only one of them having, as required by the definition, their "*stamens inserted into the receptacle:*" for, of the four officinal examples given, page 64, the first three have each a different insertion, and the fourth does not belong to this class at all: e.g. Asarum has the stamens epigynous; Canella, hypogynous; and Agrimonia, perigynous; while

Euphorbia has been shewn to be, in truth, not a dodecandrious, but a monœcious plant. Of the incorrectness of this definition, indeed, the author himself affords sufficient evidence; for, in the account of the natural order XVII. of Linnæus, Lythrum, which is a dodecandrious plant, is declared to have the "*stamens inserted into the calyx.*" (P. 96.)

In pursuing the account of the Linnæan system, we find that the XVth class, Tetradynamia, with its two orders, Siliculosa and Siliquosa, (vide pp. 50, 51,) has been, by some strange oversight, omitted.

The definitions of the orders of the XIXth class, Syngenesia, can scarcely be esteemed correct; for the diagnosis in S. Polygamia superflua, frustranea, necessaria, &c., does not depend, as here stated, on certain florets being "destitute both of stamens, and pistils," or furnished "with pistils only," or "with stamens only," but on the *imperfection* rather than the *absence* of these organs in the radius and the disk respectively, where they are found rudimentary or abortive only. The definition of S. P. segregata is unintelligible; for it is said, "This order of Syngenesia embraces such plants as bear flowers, *either simple* or *compound,* but with united tubular anthers, and with a partial calyx, all included in one general calyx." This definition would confound the orders Polygamia segregata and Monogamia, which latter is here not at all alluded to, and the flowers which it contained arranged according to the authority of those who abolished the order, in Pentandria Monogynia. A plant with a *simple* flower could not belong to Polygamia; neither could such a *simple* flower be "with a *partial* calyx, all included in one general calyx:" the postulates are incompatible.

Again, the plants in the (XL.) order Personatæ are said to be "neither much alike in general characters, or (nor?) in medical properties;" which unqualified assertion will be apt to mislead the student, for the Personatæ are fully as closely allied as most, nay, much more so than many, of the other Linnæan fragments of the natural method. But still more objectionable matter exists in certain errors which have crept into the author's definitions and illustrations of these orders; e.g. of the one just adverted to (p. 109), the "*natural characters*" are thus given: "Roots and stems indefinite; leaves obovate, cordate, ovate; *corolla four* or *five petalled;* calyx four or five parted; *stamens two to ten;* pistil one; fruit, a capsule:" whereas, on the contrary, the fact is, that the corolla will be found to be monopetalous, instead of polypetalous, and the stamens two to four, instead of ten, and generally didynamous. Malaleuca, one of the Linnæan genera affinia, does not belong to this order, but to the Myrtaceæ; yet, even did it, the definition would exclude it, as its stamens are polyadelphous. Moreover, along with Gratiola, Scrophularia, and Veronica, which are truly personate plants, the violet is associated, although it does not belong to this order at all, but to the xxixth, Campanaceæ; where it ought to have been, but is not mentioned. Nor can this be esteemed a mere casual error; for it recurs in pp. 156 and 157, where we find Viola odorata again described as belonging to the natural order Personatæ.

In conclusion, we cannot avoid expressing our regret that the author should have so continually persisted in giving to officinal plants names which have now become obsolete, and this without adding their synonymes: e.g. it should have been Alpinia (olim Elettaria) Cardamomum; Cathartocarpus (olim Cassia) Fistula; Erythrœa (olim Chironia) Centaurium; Stizolobium (olim Dolichos) Pruriens; Cetraria (olim Lichen) Islandica; Roccella tinctoria (olim Lichen Roccella), &c.

There are also several medicinal plants omitted, which, in the next edition, it would be well to insert; such as Maranta, Secale, and Acinula, Diosma, Menispermum, &c. With these alterations, the hints for which, we doubt not, the author will take in good part, this little Manual will become much more useful, by being much more correct.

